# Differentiating Temporal Plus “Insula” Epilepsy From Temporal Lobe Epilepsy by Brain Networks Based on Noninvasive Examinations

**DOI:** 10.1111/cns.70517

**Published:** 2025-07-16

**Authors:** Xu Hu, Zilin Li, Jiajie Mo, Wenhan Hu, Chao Zhang, Xiu Wang, Lin Sang, Zhong Zheng, Kai Zhang

**Affiliations:** ^1^ Department of Neurosurgery No. 904 Hospital of the PLA Joint Logistics Support Force Wuxi China; ^2^ Department of Neurosurgery Beijing Tiantan Hospital, Capital Medical University Beijing China; ^3^ Stereotactic and Functional Neurosurgery Laboratory Beijing Neurosurgical Institute, Capital Medical University Beijing China; ^4^ Department of Neurosurgery Beijing Fengtai Hospital Beijing China

**Keywords:** graphic theory, machine learning, recurrence quantification analysis, temporal lobe epilepsy, temporal plus epilepsy

## Abstract

**Aims:**

To differentiate patients with temporal plus epilepsy (TPE) with insular involvement from those with temporal lobe epilepsy (TLE) using brain network analysis based on noninvasive assessments.

**Method:**

TLE and TPE patients were retrospectively selected and matched using propensity score matching (PSM) analysis. Metabolic networks for each patient were constructed utilizing ^18^F‐fluorodeoxyglucose‐positron emission tomography, and graphic theory was applied for network analysis. Additionally, brain networks from scalp electroencephalography were assessed through recurrence quantification analysis (RQA). Graphic theoretical and RQA metrics were compared between the cohorts, and an extreme gradient boosting (XGBoost) classifier using graphic theoretical and RQA metrics was developed to differentiate TPE patients from those with TLE.

**Results:**

Twenty‐five pairs of TLE and TPE patients were selected through PSM. Among nine graphic theoretical features examined, TLE patients exhibited a significantly higher degree centrality (Dc) in the posterior insula (*p* = 0.04) and higher nodal clustering coefficients (Ncf) in both the anterior insula (*p* = 0.03) and posterior insula (*p* = 0.03), which suggested that TPE patients exhibit disrupted local connectivity and diminished integrative function in the insula, particularly in the posterior region. No significant differences were found among the 13 RQA features. Despite limited differences at the individual feature level, the XGBoost classifier achieved an accuracy of 0.77 and an AUC of 0.83, likely by capturing joint patterns across multimodal connectivity indicators.

**Conclusion:**

TLE and TPE patients demonstrate distinct brain network features, particularly Dc and Ncf within the insular region, as revealed by noninvasive assessments, which can be utilized for differentiation through machine learning algorithms.

## Graphical abstract

The classification model based on ^18^F‐FDG PET and scalp EEG brain network features effectively identifies TPE patients with insular involvement from TLE patients. There are differences in the local network features of the insular region between the two patient groups.

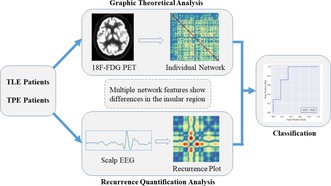

## 1 Introduction

Temporal lobe epilepsy (TLE) is the most common form of drug‐resistant epilepsy in adults that often leads to surgical intervention [1]. Anterior temporal lobectomy (ATL), involving the lateral temporal neocortical and mesial temporal structures, is the most frequently performed surgical procedure for TLE [2]. Despite advancements in ATL techniques, long‐term seizure freedom rates postsurgery remain suboptimal. Research has indicated that short‐term postoperative seizure‐free rates vary between 42% and 74% [3, 4, 5], while long‐term rates are even lower [3, 6]. A significant factor contributing to postoperative seizure recurrence is the presence of extratemporal epileptogenic zone (EZ), commonly referred to as “temporal plus epilepsy (TPE)”, often involving the insula [7]. Patients with TPE benefit less from conventional ATL, prompting a need for a tailored approach using multilobar resection guided by stereoelectroencephalography (SEEG) [8]. Therefore, it is imperative to identify TPE patients during the noninvasive preoperative evaluation phase to formulate an effective surgical strategy for improving postsurgery seizure outcomes.

Neuroimaging techniques, such as ^18^F‐fluorodeoxyglucose (FDG) positron emission tomography (PET), are widely used for the localization of EZ and examination of brain metabolic patterns. Hypometabolism has been linked to epileptogenicity [9, 10]. However, prior research has indicated that TLE may also present with hypometabolism in extratemporal regions, such as the insula [11], suggesting that hypometabolism in this region may not be a definitive diagnostic indicator of TPE. From a neurophysiological perspective, TLE and TPE are believed to reflect different underlying network dysfunctions. TLE is primarily associated with disrupted connectivity within mesial temporal structures such as the hippocampus and amygdala, while TPE involves broader extratemporal regions including the insula [12]. This widespread epileptogenic network in TPE may disrupt both local and global communication processes in the brain, affecting key functional properties such as integration, which reflects efficient long‐range communication, and segregation, which reflects local processing specialization. Thus, graphic theory, which reflects both global and regional brain connectivity through global metrics (such as small‐worldness, global efficiency, and local efficiency) and local metrics (such as clustering coefficient and degree centrality), has emerged as a prominent approach for analyzing brain network properties and has been effectively utilized to quantify their topological characteristics. The global and local features of these networks can provide insights into their overall and local nodal properties, respectively, and have been applied in exploring pathogenesis, disease classification, and predicting surgical outcomes in various neurological disorders, including Parkinson's disease, Alzheimer's disease, and epilepsy [13, 14, 15]. For instance, Aydın et al. found that the segregation and integration indices of scalp electroencephalography (EEG) in Alzheimer's disease patients were significantly decreased [16]. In a related study, the same research group also observed diminished functional brain connectivity in individuals with attention‐deficit/hyperactivity disorder compared to healthy controls and noted that this impairment could be improved with long‐term pharmacological treatment [17]. As for epilepsy, an EEG‐based study found that, compared to healthy individuals, patients with epilepsy showed a significant increase in the small‐worldness of brain networks [18]. Investigations based on the PET imaging indicated that the metabolic networks of epilepsy patients exhibited a lower assortative coefficient [19]. These findings indicate that graph theory can effectively capture pathological changes in the brain networks of individuals with epilepsy.

Scalp EEG is also a key noninvasive tool in preoperative assessments. Specific scalp EEG features, such as interictal bilateral and precentral spikes and ictal onset in anterior frontal, temporoparietal, and precentral regions, are linked to the presence of TPE [20]. However, their interpretation relies heavily on subjective analysis by neurophysiologists, which can increase clinical workload and introduce biases. Therefore, there is a pressing need to identify more objective EEG‐based indicators. Several well‐established EEG analysis methods have been proposed, each with distinct characteristics. These methods differ in various aspects, such as linear or nonlinear approaches (e.g., principal component analysis/recurrence quantification analysis [RQA]), sensitive or insensitive to volume conduction effects (e.g., inverse modeling techniques/independent component analysis [ICA]), focusing on different domains (e.g., time‐domain/frequency‐domain/time‐frequency domain), and the different consideration of causality or noncausality (e.g., granger causality analysis/coherence analysis). Among these, the concept of recurrence plays a crucial role in the dynamics of neuronal systems, and RQA has emerged as a promising method for analyzing nonlinear data in recent years [21]. RQA is predominantly utilized to describe periodic phenomena across various physical and biological contexts, including electrophysiological neuronal activity in the brain; thus, it is suitable for nonlinear, nonstationary, and short‐duration EEG analysis [22], and has been applied in several studies analyzing EEG data [23, 24].

To date, there was a lack of studies exploring the brain network differences between TLE and TPE and classifying the two epilepsy types based on these differences. Therefore, this study aimed to classify TLE and TPE using multiple brain network features derived from both PET and EEG modalities and explain the mechanisms of different brain network changes in the two types of epilepsy. Herein, personalized metabolic brain networks were developed based on PET and analyzed their graph theory characteristics. Additionally, RQA was used to analyze the network features of each patient's scalp EEG. Subsequently, the differences in these features were compared between TLE and TPE patients. Finally, several machine learning–based classifiers were developed using these features to differentiate TPE patients through noninvasive assessments.

## 2 Methods

### 2.1 Patient Selection and Grouping

A retrospective analysis was conducted on refractory epilepsy patients treated at the Epilepsy Center of Beijing Tiantan Hospital and Beijing Fengtai Hospital from January 2016 to December 2022. The inclusion criteria were as follows: (i) patients who underwent comprehensive preoperative evaluations, with high‐resolution Magnetic resonance imaging (MRI) and interictal ^18^F‐FDG PET data, (ii) those who consented to depth electrode implantation and SEEG monitoring, during which habitual seizures were recorded, (iii) seizure onset zone (SOZ) was determined by ictal SEEG, (iv) SOZ involved either the unilateral temporal lobe or temporal lobe plus adjacent structures (at least the insula), (v) PET‐MRI co‐registered images indicated a hypometabolic region confined to one hemisphere, and (vi) no history of craniotomy before SEEG.

Patients were then grouped into two groups: the TLE group, where SOZ determined by SEEG was confined to the temporal lobe, and the TPE group, where SOZ involved the temporal lobe plus insular or temporal lobe plus insula and adjacent structures. To address the unequal group sizes, a 1:1 propensity score matching (PSM) algorithm without replacement, with a caliper of 0.1, was implemented to account for the nonrandom assignment of the procedure. The propensity score was calculated using multivariable logistic regression, considering factors such as gender, SOZ side, age of seizure onset, and epilepsy duration. All patients or their guardians provided informed consent to participate. The study was approved by the Ethics Committee of the Beijing Tiantan Hospital, Capital Medical University.

### 2.2 ^18^F‐FDG PET Acquisition, Preprocessing and Group Analysis

A three‐dimensional ^18^F‐FDG PET was performed using a GE Discovery ST PET‐CT system (300 mm field of view (FOV), matrix 192 × 192, 3.27‐mm slice thickness) while the patient was awake and in a resting state. ^18^F‐FDG was administered intravenously at a dose of 3.7–5.5 MBq/kg, while PET acquisition was performed at least 6 h after any ictal event.

In the preprocessing stage, the PET images were spatially normalized to the Montreal Neurological Institute (MNI) templates and smoothed with an 8 mm full width at half maximum Gaussian filter to enhance the signal‐to‐noise ratio. Subsequently, the density of the smoothed image was normalized to the mean value of the cerebellum. Subsequently, the images of left TLE and TPE patients were flipped to analyze the right hemisphere ipsilaterally and the left brain contralateral to the SOZ.

The preprocessed PET images of the TLE group were compared with 52 healthy control (HC) subjects (males: *n* = 22, mean age: 33.8 ± 10.0 years, range: 12–45 years old) with no history of epilepsy or neurological disorders. No hypometabolism area was found using a voxel‐based, independent, two‐sample *t*‐test model, comprising age and gender as covariates. A group‐level PET T‐score map was generated, with a significant T‐score threshold set at 0.001 and a false discovery rate (FDR) correction. This methodology was similarly applied to compare the TPE group with the HC group and the TLE group with the TPE group. Preprocessing and analysis were performed using SPM 12 on MATLAB version 2019a (R2019a, Mathworks, Natick, MA, USA).

### 2.3 Metabolic Network Building and Graphic Theoretical Analysis

**FIGURE 1:**
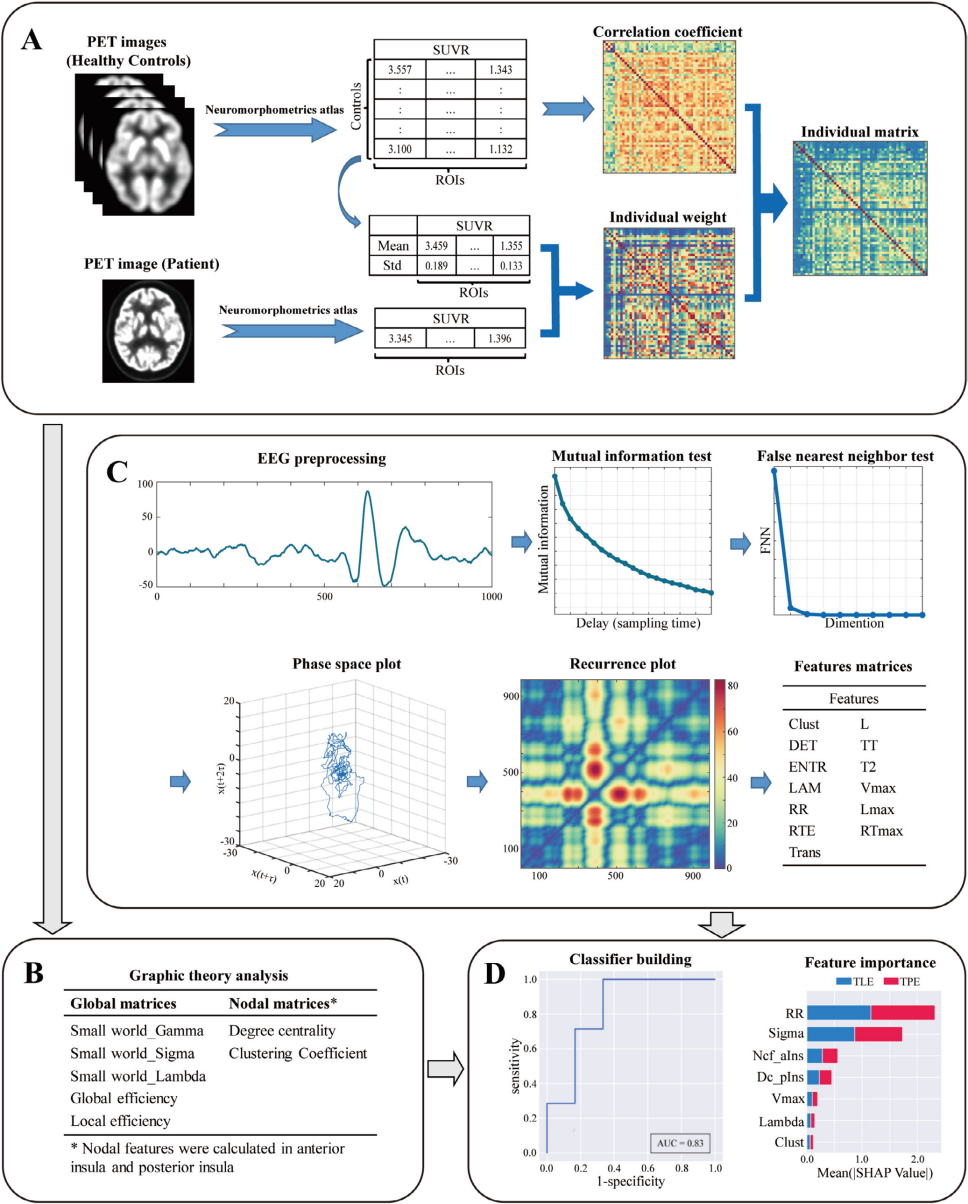
Working flow chart. (A) ^18^F‐FDG PET preprocessing and individual metabolic network construction. (B) Graphic theory analysis and features computation. (C) EEG preprocessing and recurrence quantification analysis procedure. (D) Development of the extreme gradient boosting (XGBoost) machine learning classifier.

Brain glucose metabolism values were collected from 112 distinct regions using the Neuromorphometrics atlas, excluding areas associated with the ventricular system, brainstem, white matter, and cerebrospinal fluid regions. Next, an individual‐level metabolic brain network was constructed using previously described methods [25]. A weighting matrix was calculated based on the differences in the interregional effect sizes between the average ingestion of a single subject and that of normal controls (NCs). Then the weight matrix for the individual subject was multiplied by a group‐based connectivity matrix from Pearson correlation analysis of the NC cohort, resulting in an individual metabolic brain network (Figure 1A). The matrix generated based on 56 regions of the right hemisphere was defined as the individual metabolic network, while that generated based on 56 regions of the left hemisphere was defined as the NC cohort.

For graphic theoretical analysis, small‐worldness (SW, including Sigma, Gamma, and Lambda), global efficiency, and local efficiency for each network were calculated to assess global network metrics. Moreover, the nodal clustering coefficient (Ncf) and degree centrality (Dc) were calculated for nodal network metrics in the anterior insula and posterior insula regions (Figure 1B). All parameters were evaluated across sparsity values ranging from 0.20 to 0.65, with increments of 0.05, using the Graph Theoretical Network Analysis (GRETNA) toolbox [26].

### 2.4 Scalp EEG Acquisition and RQA

The scalp EEG (EEG‐1200; Nihon Kohden Corporation, Tokyo, Japan) was recorded for all patients using the International 10–20 electrode system, with a sample rate of 1000 Hz and filter settings of 120 Hz and 0.3 Hz. EEG segments from the nonrapid eye movement (NREM) sleep phase, lasting 5–10 min, were selected for each patient based on data availability, aiming to reduce the impact of motion artifacts. Additionally, to mitigate the potential influence of seizures, data collected within 2 h before and after an ictal event were excluded from the analysis. The selected EEG segments were first visually inspected by an experienced electroencephalographer, and segments with obvious artifacts were excluded. Then, preprocessing included downsampling to 500 Hz, band‐pass filtering at 1–80 Hz, notch filtering at 50 Hz, ICA for artifact removal, and re‐referencing to the average of all channels.

The preprocessed EEG segments from each channel were subjected to RQA analysis. First, the minimum parameter τ and the lag‐delayed surrogate time series were calculated using a mutual information test with a maximum time lag of 200. Thereafter, the embedding dimension (m) was determined using the false nearest neighbor (FNN) test. Using this methodology, we established phase portraits with progressively increasing dimensions. Next, the minimum value of m that could significantly reduce the number of false neighbors was identified and chosen. The phase space of the EEG time series was plotted using the established parameters τ and m. Subsequently, a recurrence plot was created by iteratively applying a threshold function (ε) to points in the phase space, defining recurrence as the reappearance of a point in a previously occupied coordinate space [27]. Finally, various network parameters were calculated, including Clustering coefficient (Clust), Determinism (DET), Entropy of the diagonal line lengths (ENTR), Laminarity (LAM), Maximal diagonal line length (Lmax), Mean diagonal line length (L), Maximal vertical line length (Vmax), Maximal white vertical line length (RTmax), Recurrence rate (RR), Recurrence time entropy (RTE), Recurrence time of second type (T2), Trapping time (TT) and Transitivity (Trans). After excluding the first 4 s and the last 10 s of the EEG segments, a 10‐s EEG segment was randomly selected for RQA and the analysis was repeated 1000 times to obtain the average network parameters. Subsequently, the mean parameter values across all channels were calculated to serve as the feature values for each patient. The brief RQA procedure is summarized in Figure 1C.

### 2.5 Construction of the Machine Learning Classifier

A single feature matrix was developed using both the graphic theoretical analysis metrics and RQA metrics for all patients to facilitate classification. Then the feature matrix was fed into five different classifiers based on support vector machine (SVM), random forest (RF), logistic regression (LR), extreme gradient boosting (XGBoost), and naïve Bayes, each classifier employing a 10‐fold cross‐validation (CV) approach based on the data from each patient in order to ensure the stability and reliability of the results [28]. Accuracy, sensitivity, specificity, receiver operating characteristics (ROC) curve, and area under the curve (AUC) were computed to evaluate the performance of the classifier. The AUC between each classifier was compared using the Delong test [29]. TPE patients classified correctly were defined as true positive (TP) and those misclassified as false negative (FN). TLE patients classified correctly were defined as true negative (TN) and those misclassified as false positive (FP). Performance metrics were calculated as follows: accuracy = (TP + TN)/(TP + FN + TN + FP); sensitivity = TP/(TP + FN); specificity = TN/(TN + FP); precision = TP/(TP + FP); F1‐score = 2 × TP/(2 × TP + FP + FN). Furthermore, the SHapley Additive exPlanations (SHAP) method was used to analyze the significance of individual features and their interactions, thereby improving the interpretability of the machine learning decision‐making process [30, 31] (Figure 1D).

### 2.6 Statistical Analysis

Significant differences between the two patient groups were evaluated using the Student's t‐test or Wilcoxon signed‐rank test, depending on the data distribution. The normality of the data distribution was determined using the Lilliefors Test. A two‐sided *p* < 0.05 was considered statistically significant. Machine learning classification and SHAP analysis were performed using Python 3.8.8, while the PSM procedure and all statistical analysis were executed using R 4.4.1.

## 3 Results

### 3.1 Patient Characteristics and PSM Results

**FIGURE 2:**
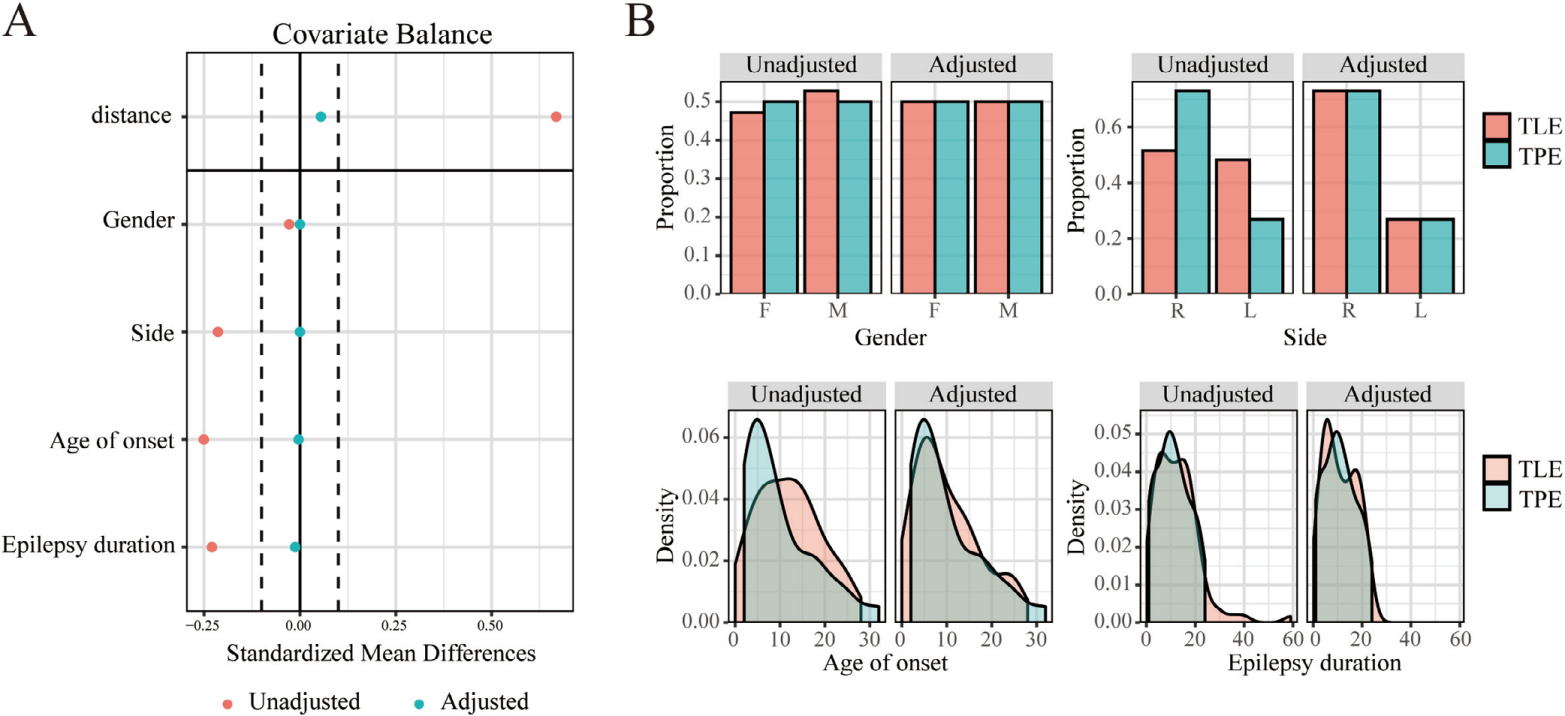
Covariate balance (A) and contribution (B) of gender, SOZ side, age of seizure onset and epilepsy duration in temporal lobe epilepsy (TLE) and temporal plus epilepsy (TPE) patients, before and after propensity score matching.

Twenty‐five patients (12 females) were included in the TPE group and 89 patients (42 females) in the TLE group. PSM with a 1:1 ratio yielded 25 matched pairs for subsequent analysis, including 25 TLE patients (13 females). PSM results are presented in Figure 2. Seven TLE patients had SOZ located in the left hemisphere and 18 in the right hemisphere. The mean age of seizure onset was 10.05 ± 7.21 years, and the mean epilepsy duration was 10.91 ± 6.83 years. Seven TPE patients experienced SOZ in the left hemisphere and 18 in the right hemisphere. The mean age of seizure onset was 9.70 ± 8.04 years, and the mean epilepsy duration was 10.90 ± 7.00 years (Table 1). The detailed information including surgical region and postoperative outcome is shown in Table S1.

**TABLE 1:** Patient clinical characteristics.

	TLE Group	TPE Group
Gender (female/male)	12/13	13/12
SOZ Side (left/right)	7/18	7/18
Age of onset	10.05 ± 7.21	9.70 ± 8.04
Epilepsy duration	10.91 ± 6.83	10.90 ± 7.00

*Note:* Data is expressed as mean ± standard deviation.

### 3.2 ^18^F‐FDG PET Group Analysis

**FIGURE 3:**
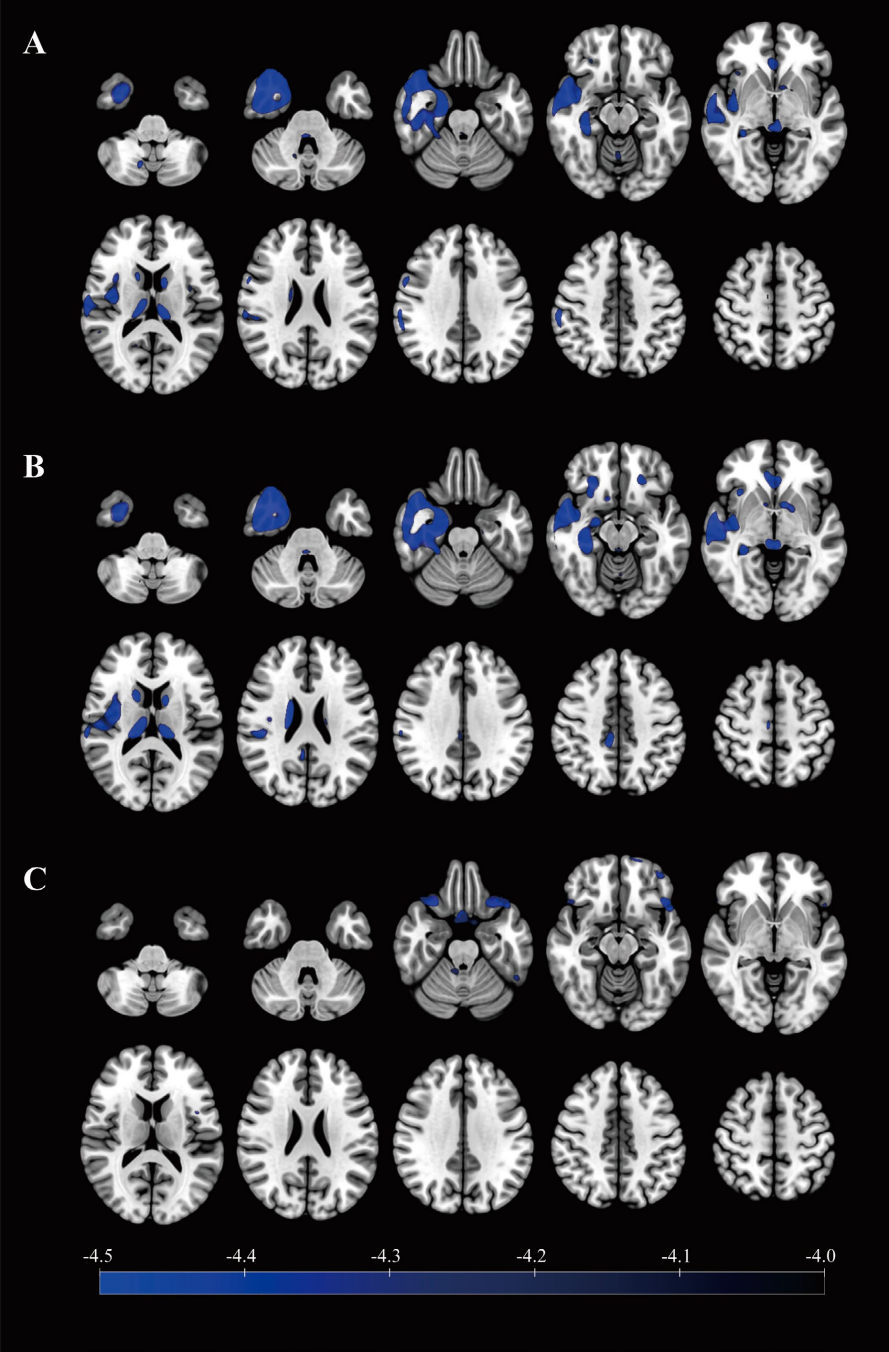
PET group analysis between (A) TLE patients and healthy controls (HC), (B) TPE patients and HC, and (C) TLE and TPE patients. Hypometabolism was mainly found in the neocortex of the temporal lobe, mesial temporal structures, insula, caudate nucleus, and thalamus in TLE and TPE patients. However, no difference in metabolic levels was found in the aforementioned regions between TLE and TPE patients.

^18^F‐FDG PET hypometabolic regions were primarily located in the neocortex of the temporal lobe, mesial temporal structures, insula, caudate nucleus, and thalamus in TLE patients (Figure 3A). Notably, it was found that the hypometabolic region in the insula was mainly located in the posterior insula. Hypometabolism was observed in similar regions in TPE patients (Figure 3B). No significant metabolic differences were observed between TLE and TPE patients in the abovementioned regions (Figure 3C).

### 3.3 Graphic Theoretical Analysis and RQA Features

**FIGURE 4:**
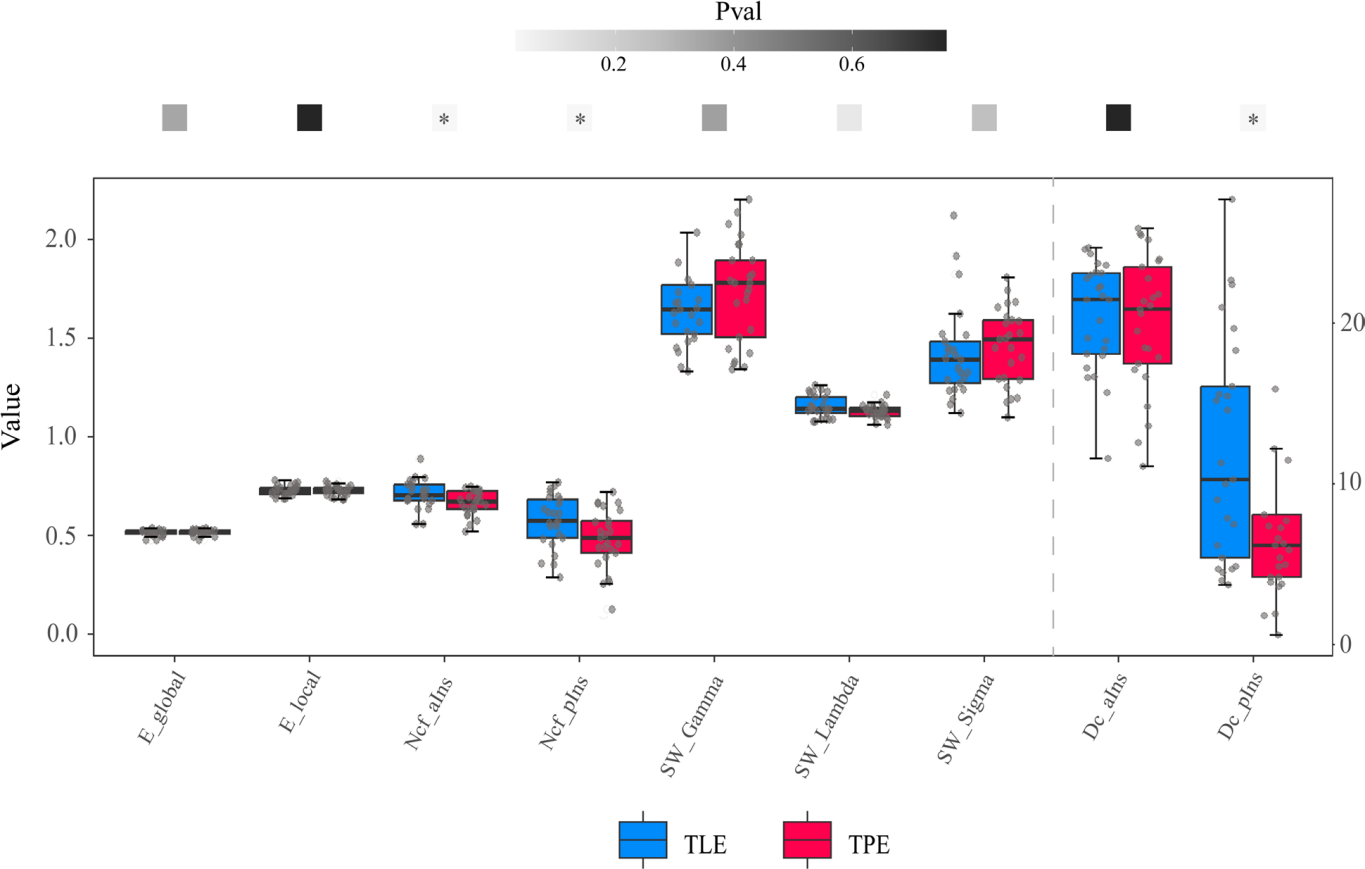
Graphic theoretical features. The nodal clustering coefficient of the anterior and posterior insula and degree centrality of the posterior insula differed significantly between TLE and TPE patients. aIns, anterior insular; Dc, degree centrality; E_global; global efficiency; E_local, local efficiency; Ncf, nodal clustering coefficient; pIns, posterior insular; SW, small world.

None of the features passed the normality test; therefore, the Wilcoxon signed‐rank test was used for group comparisons. For graphic theoretical features, no significant differences were found between the two groups in SW (*p* = 0.36 for Gamma, *p* = 0.26 for Sigma, *p* = 0.09 for Lambda), global efficiency (*p* = 0.34), local efficiency (*p* = 0.76) and Dc in the anterior insula (*p* = 0.78). However, the Dc of the posterior insula and Ncf of the anterior and posterior insula were higher in the TLE group than in the TPE group (*p* = 0.04 for Dc in the posterior insula and *p* = 0.03 for Ncf in the anterior and posterior insula). The results of the graphic theoretical analysis are depicted in Figure 4.

**FIGURE 5:**
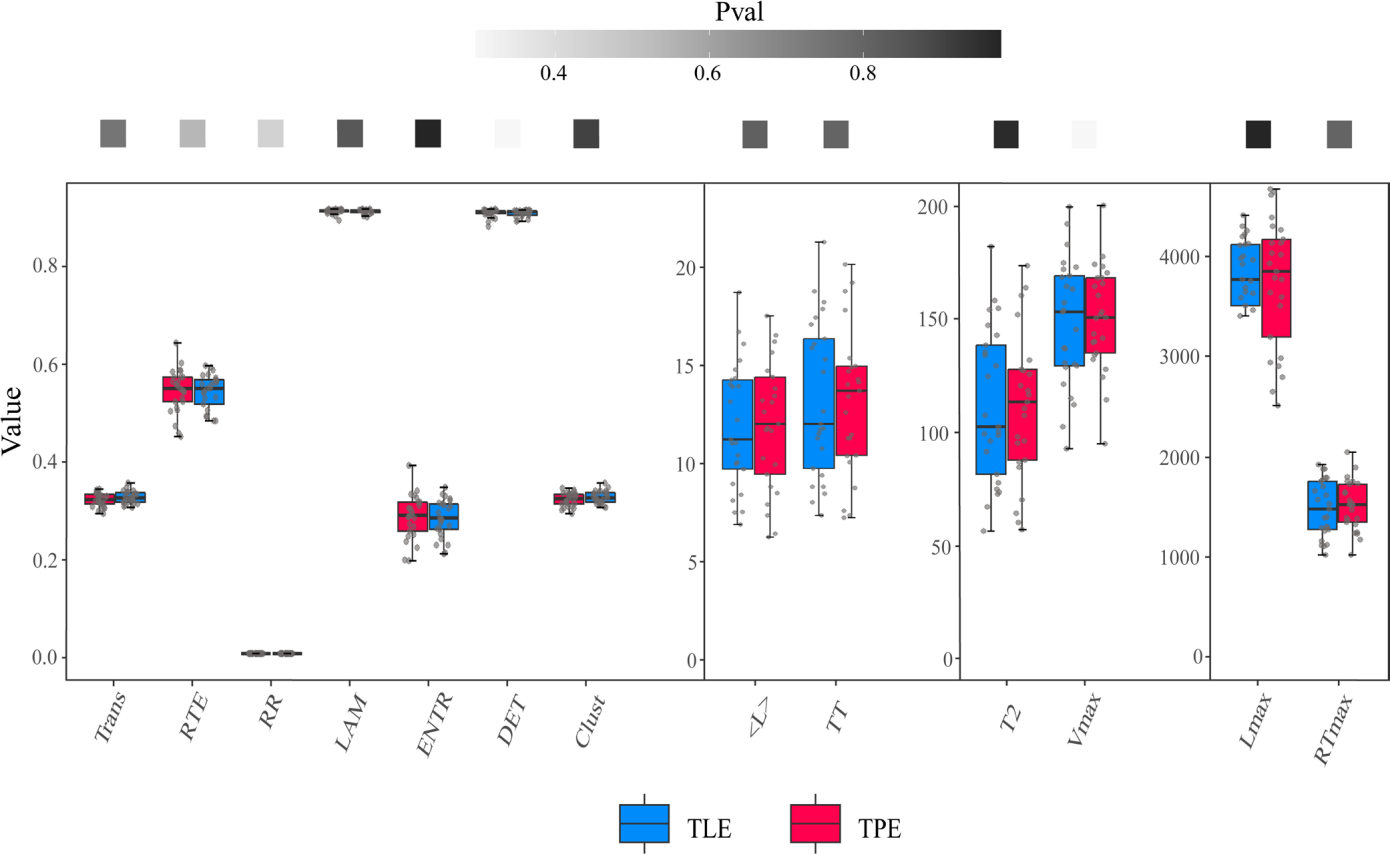
Recurrence quantification analysis (RQA). No significant difference was found between TLE and TPE patients regarding RQA features. Clust, clustering coefficient; DET, determinism; ENTR, entropy of the diagonal line lengths; L, mean diagonal line length; LAM, laminarity; Lmax, maximal diagonal line length; RR, recurrence rate; RTE, recurrence time entropy; RTmax, maximal white vertical line length; Trans, transitivity; TT, trapping time; T2, recurrence time of second type; Vmax, maximal vertical line length.

However, no significant differences were found between TLE and TPE groups in all 13 RQA features (*p* = 0.44 for RR, *p* = 1.00 for DET, LAM and TT, *p* = 0.86 for ENTR, *p* = 0.83 for RTE, *p* = 0.30 for Clust and Trans, *p* = 0.55 for Lmax, *p* = 0.82 for RTmax and Vmax, *p* = 0.98 for T2, *p* = 0.92 for L). The results of RQA are displayed in Figure 5.

### 3.4 Performance of the Feature‐Based Classifier

**FIGURE 6:**
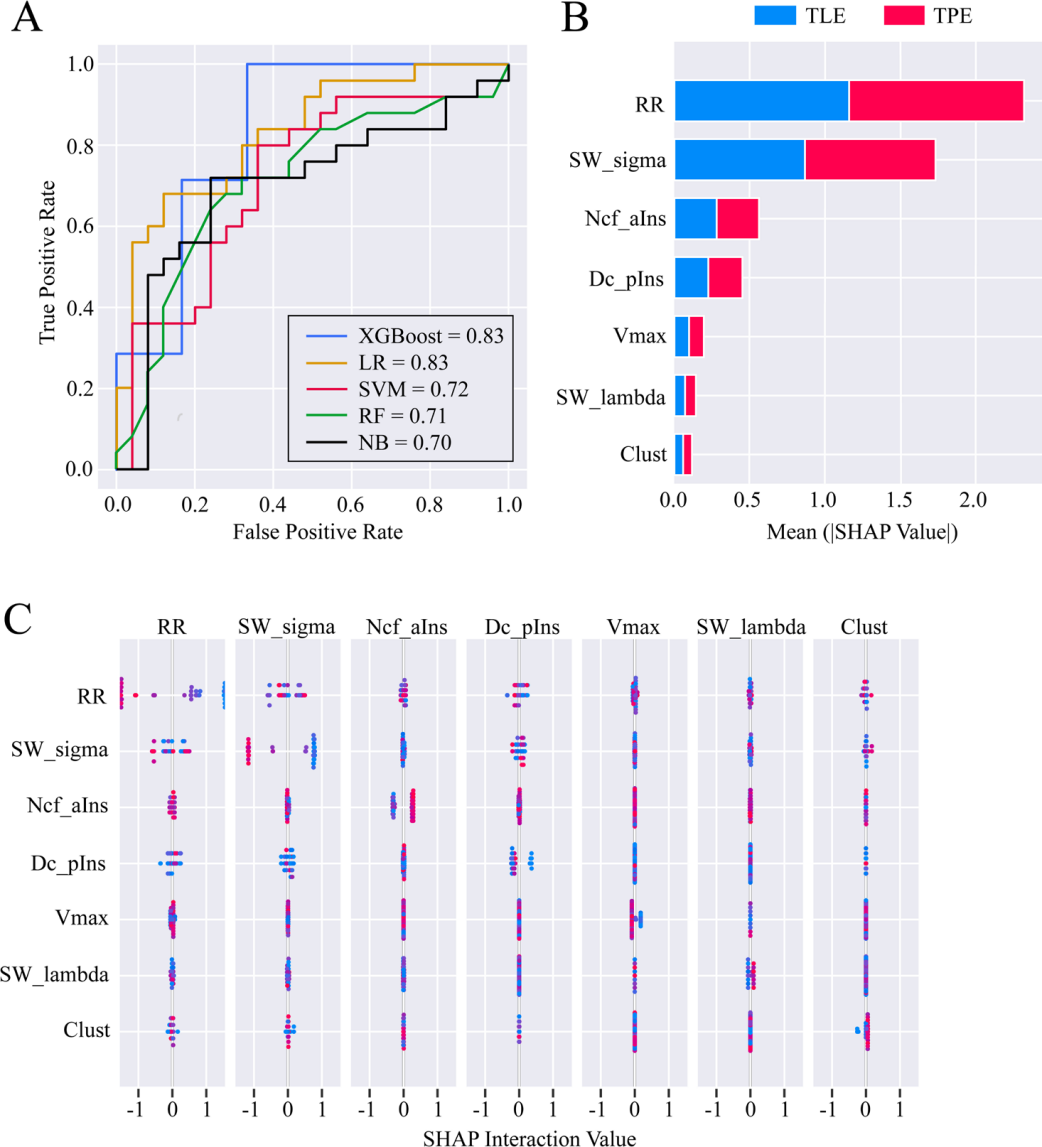
Performance of the classifiers. (A) The classifiers' areas under the curves for algorithms XGBoost, logistic regression, supporting vector machine, random forest, and naïve Bayes were 0.83, 0.83, 0.72, 0.71, and 0.70. (B) Feature importance based on SHapley Additive exPlanations (SHAP) of the XGBoost‐based classifier showed that recurrence rate (RR) and sigma played important roles in classification. (C) Interaction effect analysis based on SHAP analysis of the XGBoost‐based classifier showed a certain interaction effect between RR and sigma. aIns, anterior insular; AUC, area under the curve; Clust, clustering coefficient; DC, degree centrality; LR, logistic regression; NB, naïve Bayes; Ncf, nodal clustering coefficient; pIns, posterior insular; RF, random forest; RR, recurrence rate; SVM, supporting vector machine; SW, small world; TLE, temporal lobe epilepsy; TPE, temporal plus epilepsy; Vmax, maximal vertical line length; XGBoost, extreme gradient boosting.

A 50 × 22 feature matrix containing nine graphic theoretical features and 13 RQA features of each patient was built and then the combined feature matrix was fed into five machine learning‐based classifiers. After CV, the AUC of the XGBoost‐based classifier was significantly higher than that of the other three classifiers except for the logistic regression‐based classifier (all *p* < 0.01, Figure 6A). Considering that the XGBoost classifier also outperformed the logistic regression classifier in terms of accuracy and F1‐score, we ultimately selected the XGBoost classifier for further SHAP analysis. The XGBoost classifier achieved an accuracy, sensitivity, specificity, precision, F1‐score, and AUC of 0.77, 0.80, 0.75, 0.77, 0.78, and 0.83, respectively, and the corresponding metrics for the other four classifiers were presented in Table S2. SHAP analysis revealed that RR, Sigma, Ncf of anterior insula, and Dc of posterior insula had greater contributions to the classifier (Figure 6B). Furthermore, the interaction effect analysis identified a certain interaction effect between RR and Sigma, whereas no significant interaction effects were observed between other features (Figure 6C).

## 4 Discussion

The present study developed a classifier based on noninvasive preoperative evaluation tests for differentiating TPE patients with insular involvement from those with TLE, thereby guiding clinical diagnosis and treatment. First, metabolic differences between the two groups were analyzed using 18F‐FDG PET. Then, graphic theoretical features derived from individual metabolic networks and RQA features from the scalp EEG were calculated and compared between the patient groups. Finally, a classifier based on the XGBoost algorithm was constructed to distinguish between the two groups, and feature importance and interaction effects were evaluated.

Hypometabolism in PET has been associated with the presence of EZ [9, 10], with hypometabolism in the insula possibly indicating the presence of TPE. However, the irritative zone or functional deficit regions can also present as hypometabolic [32, 33], making it difficult to distinguish between TPE and TLE based solely on the presence of hypometabolism. This study found that TLE patients experienced hypometabolism in the insular region, similar to TPE patients with insular involvement. This suggests that PET alone is not a reliable method for accurate classification. Distinguishing between these epilepsy forms relies more on anatomical, electrophysiological, and semiological characteristics [20].

Previous research suggests that patient semiology and scalp EEG findings can help differentiate between TPE and TLE [20]. However, due to varying levels of expertise among epileptologists, developing objective classifiers is crucial. Graphic theoretical analysis utilizing metabolic networks has been applied in studying various neurological disorders [19, 34, 35] and was therefore included in our feature set to capture disease‐related topological alterations. Although no significant differences in global network features were found between TPE and TLE, TPE patients tended to show increased small‐worldness. This may reflect more complex and spatially widespread epileptogenic networks, as enhanced small‐worldness is generally linked to greater disease severity or a more widespread extent of abnormal network activity [36, 37], which aligns with the larger EZs observed in TPE patients. In contrast, nodal‐level analysis revealed more specific dysfunctions. A significant reduction in Ncf of the entire insula and Dc of the posterior insula was noted in TPE patients, which contrasts with previous fMRI studies where Ncf typically increased among epilepsy patients compared with HCs [38]. Thus, these findings showed a loss of local connectivity and integrative capacity in these regions, which might reflect network node damage or disconnection due to direct EZ involvement in TPE patients. This suggests that, although hypometabolic levels in the insula were not significantly different between TLE and TPE patients, variations in nodal connectivity in the network were detected. Notably, the posterior insula was more prominently involved than the anterior insula, aligning with the results from the PET group analysis. Therefore, the dysfunction in the posterior insula might be a critical factor distinguishing TPE from TLE. In a word, the impaired insular connectivity—particularly in the posterior region—underlies the observed network‐level differences between the two conditions, and alterations were not solely reflected in metabolic intensity but were also detectable through topological analysis, highlighting the added value of graph‐theoretical measures in identifying subtle, region‐specific disruptions.

Scalp EEG is a crucial component in preoperative assessments. RQA using neurophysiological data has been employed to investigate alterations in network dynamics associated with neurological diseases [24, 39]. Generally, elevated RQA features signify enhanced internal functional connectivity and improved information exchange in the brain network [24]. However, the present study found no significant differences in RQA features between TLE and TPE patients. This suggested that despite the notable differences in certain scalp EEG features between TPE and TLE patients [20], EEG brain networks were comparable between the two cohorts. Given that scalp EEG‐based network analysis may be influenced by factors such as artifacts or signal attenuation, future analyses utilizing intracranial EEG may yield a more precise insight into the specific brain network alterations distinguishing TLE from TPE patients.

The XGBoost classifier, incorporating both graphic theoretical and RQA features, effectively distinguished between TLE and TPE patients. This suggests that even in the absence of consistently significant findings from group‐level analysis, features obtained through noninvasive assessments may still hold value as potential biomarkers for classification purposes. A notable advantage of SHAP analysis, which visualizes machine learning processes, lies in its capacity to intuitively assess the contribution of each feature to model predictions, thereby circumventing the limitations associated with the “black box” nature of conventional models [30, 31]. In the feature importance analysis based on SHAP, higher rankings for RR, small‐worldness, Ncf of the anterior insula, and Dc of the posterior insula were identified. As mentioned earlier, these graphic theoretical features differed between the two groups, highlighting distinct states of the metabolic network. Among the RQA features, RR is an efficient indicator of the regularity or predictability of a given time series [39]. This suggests that although no differences were observed in the group analysis, there were still underlying differences in the complexity of scalp EEG networks between the two groups, identifiable through the machine learning process. It also demonstrated that the brain network metrics based on RQA could classify the two patient groups, confirming the scientific validity of this method. SHAP was also used to explore interactions among features of the same modality and found no significant interactions between the most impactful graphic theoretical and RQA features. Specifically, a potential interaction effect between RR and sigma was observed. Although both metrics reflect aspects of brain network complexity—suggesting a possible underlying correlation—the fact that they derive from different modalities (PET and scalp EEG) led us to conclude that this interaction is more likely an artifact of the machine learning algorithm rather than a genuine representation of the relationship between metabolic and EEG networks.

Nevertheless, this study has several limitations. First, the classification of TLE and TPE relied solely on the SOZ identified through ictal SEEG, without considering surgical and prognostic data, which may have resulted in the misclassification of some patients. Second, the sample size in this study was relatively small due to the inclusion criteria and the limited number of TPE patients, potentially leading to unreliable group analyses or classifier overfitting. Besides, due to some patients being early cases, their awake EEG data had significant artifacts, and even after artifact removal using ICA, the classification performance was still suboptimal. Therefore, sleep phase EEG was ultimately used for RQA and classification, which may introduce potential bias into the results. Also, only RQA was used for EEG analysis, and given the diversity of EEG analysis methods, it remains to be seen whether analysis based on other methods could yield similarly good classification results. Future studies should include larger studies incorporating cohorts with preoperative data, surgical approaches, and postoperative follow‐up as well as more diverse brain network analysis methods based on neuroimaging or EEG to enhance the accuracy and reliability of TLE and TPE patient classification.

## 5 Conclusion

In summary, this study explored the differences in graphic theoretical features derived from PET metabolic networks and RQA features obtained from scalp EEG in TLE and TPE patients. A machine learning‐based classifier was developed utilizing these characteristics to distinguish between the two patient groups. This classifier can potentially guide treatment strategies for TPE patients in clinical settings and improve their surgical outcomes.

## Author Contributions

**Xu Hu:** writing – original draft, writing – review and editing, data curation, methodology, validation, visualization. **Zilin Li:** writing – original draft, writing – review and editing, methodology, software, validation, and visualization. **Jiajie Mo:** writing – review and editing, methodology, and software. **Wenhan Hu:** writing – review and editing, data curation, and funding acquisition. **Chao Zhang:** writing – review and editing and data curation. **Xiu Wang:** writing – review and editing, data curation, and funding acquisition. **Lin Sang:** writing – review and editing and data curation. **Zhong Zheng:** writing – review and editing and data curation. **Kai Zhang:** conceptualization, resources, writing – review and editing, funding acquisition, supervision, data curation, and investigation.

## Consent

All the patients provided informed consent for the use of their medical records.

## Conflicts of Interest

The authors declare no conflicts of interest.

## Supporting information


Data S1.

## Data Availability

The data that support the findings of this study are available from the corresponding author upon reasonable request.
