# Supplementary material for: Differentiating Temporal Plus “Insula” Epilepsy From Temporal Lobe Epilepsy by Brain Networks Based on Noninvasive Examinations
Source: CNS Neurosci Ther. 2025 Jul 16;31(7):e70517. doi: 10.1111/cns.70517 (PMC12264451; doi:10.1111/cns.70517)
Supplement: Supplementary file 1 — Data S1. [file CNS-31-e70517-s001.docx]

**Supporting Information**

**Table S1** Clinical information of included patients

| Patient ID | Gender | Age of onset | Epilepsy duration | SOZ side | Resection region^*^ | Outcome/  ILAE^*^ |
| --- | --- | --- | --- | --- | --- | --- |
| 1 | F | 24 | 5 | L | MT | 1 |
| 2 | F | 24.75 | 0.25 | R | MT | 1 |
| 3 | F | 0.5 | 17.5 | L | LT+MT | 1 |
| 4 | M | 8 | 6 | L | LT+MT | 1 |
| 5 | M | 13 | 6 | R | LT+MT | 1 |
| 6 | M | 2 | 23 | R | LT+MT | 1 |
| 7 | F | 18 | 10 | R | MT | 1 |
| 8 | M | 17 | 7 | R | LT+MT | 1 |
| 9 | M | 15 | 16 | R | LT+MT | 1 |
| 10 | M | 24 | 6 | R | LT+MT | 3 |
| 11 | F | 5 | 9 | R | LT+MT | 1 |
| 12 | M | 4 | 21 | R | LT+MT | 1 |
| 13 | M | 4 | 21 | L | LT+MT | 1 |
| 14 | M | 3 | 17 | R | LT+MT | 5 |
| 15 | F | 7 | 5 | L | LT+MT | 1 |
| 16 | M | 11 | 12 | R | LT+MT | 1 |
| 17 | F | 12 | 12 | R | LT+MT | 1 |
| 18 | M | 6 | 19 | R | LT+MT | 3 |
| 19 | M | 14 | 11 | R | LT+MT | 1 |
| 20 | F | 13 | 3 | R | LT+MT | 1 |
| 21 | F | 2 | 2 | L | LT+MT | 3 |
| 22 | F | 7 | 16 | R | LT+MT | 1 |
| 23 | F | 5 | 19 | R | LT+MT | 2 |
| 24 | M | 7 | 3 | R | LT+MT | 1 |
| 25 | F | 5 | 6 | L | LT+MT | 1 |
| 26 | M | 4 | 6 | R | LT+MT+I | 1 |
| 27 | F | 5 | 20 | R | LT+MT+I | 4 |
| 28 | F | 2 | 16 | L | LT+MT+I | 1 |
| 29 | M | 3 | 24 | L | NA | NA |
| 30 | M | 17 | 7 | R | LT+MT+I+OFC | 1 |
| 31 | M | 3 | 21 | R | LT+MT+I | 1 |
| 32 | F | 11 | 12 | R | LT+MT+I | 1 |
| 33 | F | 3 | 9 | R | LT+MT+I | 1 |
| 34 | M | 6 | 17 | R | NA | NA |
| 35 | M | 32 | 3 | R | LT+MT+I | 3 |
| 36 | M | 4 | 1 | L | LT+MT+I | 1 |
| 37 | F | 24 | 2 | R | LT+MT+I | 1 |
| 38 | F | 24 | 9 | L | NA | NA |
| 39 | M | 18 | 8 | R | LT+MT+I | 1 |
| 40 | F | 18 | 13 | R | LT+MT+I | 1 |
| 41 | F | 10 | 2 | L | LT+MT | 4 |
| 42 | M | 8 | 3 | L | LT+MT | 3 |
| 43 | M | 3 | 22 | R | LT+MT+I | 1 |
| 44 | F | 6 | 15 | R | NA | NA |
| 45 | F | 8.5 | 1.5 | R | LT+MT+I | 2 |
| 46 | F | 4 | 9 | R | NA | NA |
| 47 | F | 6 | 19 | R | LT+MT+I | 1 |
| 48 | F | 3 | 8 | L | LT+MT+I | 1 |
| 49 | M | 11 | 11 | R | LT+MT+I | 1 |
| 50 | M | 9 | 14 | R | NA | NA |

TLE group: patient 1-25, TPE group: patient 26-50

F, female, I, insula, L, left, LT, lateral temporal lobe, M, male, MT, mesial temporal lobe, NA, not available, OFC, orbitofrontal cortex, R, right

* Patients 29, 34, 38, 44, 46 and 50 refused to undergo resection surgery after SEEG implantation due to potential surgical complications.

**Table S2** Classification performance results of different classifiers

| Classifier | Accuracy | Sensitivity | Specificity | Precision | F1-score | AUC |
| --- | --- | --- | --- | --- | --- | --- |
| SVM | 0.66 | 0.60 | 0.72 | 0.68 | 0.64 | 0.72 |
| RF | 0.70 | 0.72 | 0.68 | 0.69 | 0.71 | 0.71 |
| LR | 0.72 | 0.72 | 0.72 | 0.72 | 0.72 | 0.83 |
| XGBoost | 0.77 | 0.80 | 0.75 | 0.77 | 0.78 | 0.83 |
| NB | 0.70 | 0.64 | 0.76 | 0.73 | 0.68 | 0.70 |

AUC, area under the curve, LR, logistic regression, NB, naïve Bayes, RF, random forest, SVM, supporting vector machine, XGBoost, extreme gradient boosting.
